# Supplementary material for: Are carbon emissions trading and green financial instruments synergistic? -Comprehensive quantitative research based on content analysis
Source: PLoS One. 2024 Mar 7;19(3):e0298601. doi: 10.1371/journal.pone.0298601 (PMC10919601; doi:10.1371/journal.pone.0298601)
Supplement: S1 Data — (ZIP) [file pone.0298601.s001.zip › Data/地级市Z_F工作报告.zip!/─■╧─/╬Γ╓╥╩╨/└┤╫╘╣1⁄2╓┌║┼í╛┬φ┐╦╩2╛▌═°í┐_2003.docx]

各位代表:

现在，我代表市人民政府向大会作政府工作报告，请予审议，并请市政协委员提出意见。

一、2002年工作的回顾和今年工作的目标任务

2002年，是我市经济社会发展中极为重要的一年。面对入世后国际经济变化起伏，国内经济激烈竞争的复杂形势，全市各族人民在自治区党委、政府和市委的正确领导下，高举邓小平理论伟大旗帜，努力实践“三个代表”重要思想，深入贯彻党的十六大精神和自治区第九次党代会精神，扭住发展不放松，深化改革不懈怠，团结奋发，真抓实干，使国民经济继续保持快速发展，社会各项事业长足进步，较好地完成了年度各项目标任务。

——市域经济快速发展。全市完成国内生产总值96亿元，增长11%，其中第一产业完成增加值20.5亿元，增长3.4%；第二产业完成增加值49.5亿元，增长15.5%；第三产业完成增加值26亿元，增长10%。全市地方财政收入完成5.2亿元，可比增长19.8%；城乡居民储蓄存款余额92.2亿元，比年初增长8.4%，金融运行平稳，城乡消费稳中趋旺，经济内部活力增长。

——工业经济持续攀升。深入推进了工业结构调整、企业改革和技改扩建，骨干企业、优势产业逐步扩大。美利纸业2640纸机，宇华纸业1760纸机，西夏、宏威、华泰鑫公司3个30万吨干法悬窑水泥，香山酒业10000吨枸杞酒，夏进50000吨无菌奶扩建等项目建成投产。开工建设工业治污项目34个，工业企业达标排放率达93.7%。工业经济总体进入一个新的成长期，增速和效益继续在全区四个地市中保持领先位置。全年辖区工业完成总产值140亿元，同比增长18.3%，工业对国内生产总值增长的贡献率达到52%。

——农业结构调整活力显现。以吴忠“国家农业科技园区”为重点，投资6678万元，新建科技示范园区70多个；引进了丹麦英特尔等10多家公司参与农业产业化经营；通过市场牵引、龙头带动、技术支撑，促进农业和农村经济结构调整，农业产业提升进入了一个新的转型期，养殖业、设施农业和“三白一红一绿”特色产业优势明显，其中畜牧业在农业总产值中的比重达到48%。

——城乡基础设施建设步伐加快。全社会固定资产投资完成43.8亿元，增长12.1%。吴忠市区和六县(市)城镇建设进入了一个前所未有的活跃期，中卫、中宁、青铜峡、盐池、同心等市县城市建设力度空前加大，面貌一新。市区顺利实施了“居民计划”、新区广场、开元大道、柴渠路等工程和一批基础设施建设项目，全市城镇化水平稳步提高。石中高速公路建成通车，中郝公路及沙坡头水利枢纽工程建设加快。吴青公路正式开工建设，县乡公路建设投资1.9亿元。城乡电网改造、公众电信网扩容工程进展顺利，基础设施条件进一步完善。

——对外开放取得新成效。全市上下紧紧抓住国家实施西部大开发战略的历史机遇，积极主动地争取项目和资金。据不完全统计，全市共争取投资百万元以上项目103个，涉及资金10.48亿元，有力推动了地方经济发展。以承办“中国宁夏(吴忠)民间艺术游”、“大漠黄河旅游节”、“宁夏枸杞节”等活动为载体，抓住鲁宁、闽宁合作的契机，扩大对外宣传和交流合作。年内共签订招商引资项目51个，合同引进资金4.5亿元。

——人民生活水平稳步提高。农民人均纯收入达到2460元，增长5%。农民税费改革试点全面推开，有效地减轻了农民负担。加强山区扶贫开发，实现了山区农业丰收，山区群众生产生活条件明显改善。城镇居民可支配收入达到5600元，增长9.8%。社会保障工作深入推进，养老保险、失业保险基金征缴率大幅提高，覆盖面不断扩大，城市低保、城镇职工基本医疗保险制度改革稳步进行，对社会弱势群体的保障能力有所增强。

——社会事业全面发展。加快推进了科技兴农和工业技术创新，科技对经济的支撑力不强。实施教育结构调整，川区巩固提高了“普九”成果，山区基本扫除青壮年文盲。职业教育、民族教育有了新发展。广泛开展了群众性文化活动和全民健身、公民无偿献血等活动，文化、体育、卫生事业进一步加强。计划生育工作全面完成了既定的任务指标。广播电视、新闻出版、防震减灾、老龄、残疾人等事业深入发展。民族团结不断增强，“双拥”和军民警民共建活动向纵深推进，军政、军民关系更加密切。

——民主法制建设不断加强。认真执行人民代表大会及其常委会的决议，加强与人民政协、各民主党派和人民团体的联系。坚持依法治市和依法行政，全面落实行政执法责任制，启动了吴忠市政务大厅。推进了村务公开、厂务公开、政务公开，基层民主制度建设取得新的进展。加强财政审计监督和纠风工作，有效预防和治理违纪违规现象。深入实施“四五”普法，加强社会治安综合治理，开展治爆缉枪、禁毒追逃等“严打”整治专项斗争，严厉打击了各种违法犯罪活动，维护了社会稳定。

各位代表，过去的一年，全市经济快速发展，城乡面貌显著变化，人民生活不断改善，各条战线生机勃勃，各项工作成效明显。取得这些来之不易的成绩，一是坚定不移地贯彻了中央、自治区重大决策和市委的工作部署，有针对性地采取了一系列切实可行的措施，创造性地开展了工作。二是勇于自加压力，确立了务实求进的奋斗目标和工作重点，激发了调动了全市各族人民加快发展的热情。三是统揽全局、抓住关键，认真解决前进中的困难和问题，保证了各项工作顺利推进。四是有效履行了政府职能，促进了各级政府部门加快节奏，提高效能，狠抓落实，以全新的工作作风开创了新的工作局面。这些成就的取得，是中央、自治区和市委正确领导、大力支持的结果，是全市各族人民团结奋发、务实苦干的结果，也是市人大及其常委会有效监督，人民政协关心帮助和方方面面协助配合的结果。在此，我代表市人民政府，向工作在各条战线上的工人、农民、知识分子和干部，向关心支持政府工作的人大代表、政协委员，向各民主党派、工商联、人民团体和社会各界人士，向驻吴部队、武警官兵和公安政法干警致以崇高的敬意和衷心的感谢！

总结一年的工作，我们也清醒地看到，全市经济和社会发展中还存在不少矛盾和问题。主要是：经济结构性矛盾对经济发展的制约依然比较突出；市财力薄弱，；基础设施建设比较滞后；企业改制的成本和难度加大，部分企业机制转换滞后，经营困难；就业和再就业压力增大；农民收入增长比较缓慢；一些地方和部门职能转变还未完全到位，尤其在思想观念转变和机制、制度创新上，在管理经济的理念和方式上，与先进地区相比还存在较大差距，市场经济秩序和总体发展环境仍然不够理想；精神文明建设和民主法治建设还存在一些薄弱环节。这些都需要我们采取更加有效的措施，认真加以解决。

2003年是贯彻落实党的十六大精神、全面建设小康社会的新一年。面对新的形式和任务，我们必须准确把握国内外经济发展的变化和趋势，紧跟全国各地竞争激烈、加快发展的步伐，坚定信心，奋发有为，集中全力开拓经济和社会发展的新局面。总体要求是:以邓小平理论和“三个代表”重要思想为指导，深入贯彻党的十六大和自治区第九次党代会精神，解放思想，更新观念，加强管理，创新经济发展环境，加快经济结构调整，全面深化改革开放，大力推进工业化、城市化和农业产业化，推进经济跨越式发展；不断完善社会保障体系，努力扩大就业，稳步提高城乡人民生活水平；切实加强政治文明和精神文明建设，促进社会繁荣进步，为全面建设小康社会扎实基础。经济和社会发展的主要预期目标是：国内生产总值力争达到107亿元，增长10%以上，其中第一产业增长4.5%，第二产业增长15%，第三产业增长10%；全社会固定资产投资50亿元以上，增长15%；地方财政收入5亿元，可比增长10%；城镇居民人均可支配收入5930元，增长6%；农民人均纯收入2550元，增长4%；城镇登记失业率控制在5%以内，人口自然增长率控制在11.5‰以内。

二、努力推动经济跨越式发展，加快全面建设小康社会进程

在我市目前的发展水平和现实基础之上，全面建设小康社会，必须付出艰辛的努力。去年全市人均国内生产总值640美元，要达到2020年人均国内生产总值3000美元的目标，差距很大，任重道远，我们必须以勇于争先、敢于超越的胆略和气魄，以超常规的发展思路干超常规的事业；必须树立强烈的忧患意识、危机意识，坚定大发展、快发展的信心和决心，紧紧抓住发展这个第一要务，举全市之力，坚定不移地推进工业化、城市化和农业产业化，坚持不懈地优化创新经济发展环境，为市域经济的发展，为建设繁荣、昌盛、文明的新吴忠艰苦风斗。

(一)突出产业升级和项目带动，大力推进新型工业化

加快推进工业化，是促进我市经济快速发展，尽快缩小与先进地区差距的重大战略选择。如果不走新型工业化的道路，就难以创造更快的发展速度，形成更强的竞争优势，聚集更雄厚的财力物力，全面建设小康社会就是一句空话。推进新型工业化，就要全面贯彻自治区加快工业化进程工作会议精神和市委“稳住一块、新增一块、开拓一块”的总体部署，从吴忠的实际出发，依托现有资源、产业基础和市场条件，在培育高新技术产业的同时，突出抓好传统产业升级、项目带动和非公有制经济发展。集中力量突破技术、信息、资金、人才、体制等方面的“瓶颈”制约，稳固基础，挖掘潜力，推动工业经济优化升级。不断提升内涵。立足保持现有企业稳定增长态势，继续狠抓企业技改、内部管理、产品质量、市场服务等环节，加快采用信息与高新技术改造提升化工、冶金、乳品、造纸、建材等产业，研究开发新产品，加速产品升级换代。优化配置资本、技术、资源等生产要素，争创名优品牌，培育锻造大企业、大公司、大集团，增强产业集中度和企业竞争力。全力扩张外延。把园区建设作为加快工业发展的重要突破口，明确目标责任，制定优惠政策，实行封闭管理，加快基础设施建设，力争在项目进园区上有突破性进展。市工业园区力争完成一期工程的开发建设，吸引更多的企业、项目进园发展。支持县(市、区)加快利通金积、中卫东园、中宁石空及青铜峡、同心工业园区建设，打造工业发展平台，增强工业发展后劲。通过抓项目和投资扩张工业经济总量，在抓好全世达6000吨金属镁、丰硕公司5000万平方米土工布等项目的同时，争取新上一批大的生产项目，尤其是在农副产品加工企业的新增扩大上，务求有所突破。积极创新机制。按照建立现代企业制度的要求，深化企业改制，通过改组、破产、兼并、股份制改造等形式，加快企业资产重组，明晰产权、责权关系，完善法人治理结构，建立有效的企业经营者激励约束机制，激活企业经营。进一步打破体制和机制约束，毫不动摇地支持、鼓励非公有制经济发展，支持、鼓励非公有制经济参与国有、集体企业改组改造，提高非公有制经济在地方经济总量中的比重。创造良好环境。全力优化工业发展环境，对列入全区20强的6家大型企业及全市30户骨干企业加强保护和服务，并重视和支持其他企业快速发展。采取有力措施，杜绝对企业乱摊派、乱收费和不合理的检查评比、达标验收。全面整顿市场秩序，完善信用体系，强化安全生产，保障工业稳定发展。

(二)加快推进城市化，统筹城乡发展。我市要在新一轮结构大调整、经济大发展、市场大竞争中抢占先机、有所作为，必须重视和处理好城市化与工业化、农业产业化的互动、互利关系，坚持以城市发展带动经济发展，加快推进城市化进程。加强规划和管理。把城市作为一种资源、一种生产要素、一份重要资产来进行规划和管理，促进生产要素合理配置，增强城市的聚集、辐射效应。修订完善城市发展规划，做好市区、各县市城市和小城镇的规划制定和修编，做好城市重点发展区域的详细规划，严格按照规划建设和管理城镇。多渠道增加城市投资。充分发挥政府资金、国债资金的引导作用，按照市场化运作和谁投资、谁经营、谁受益的原则，采取合资、合作、独资等多种形式，鼓励、吸引私人资本、社会资本、外来资本参与城市建设。加大政策引导力度，采取土地资本运作及出让经营权、收益权、冠名权、股权等形式，盘活城市资产，聚集建设资金，激活城市开发经营。加速扩张城市规模。按照全市城市化发展目标，突出特色和定位，加快推进各市县城市建设和中心集镇建设。着力盘活城市资源，积极筹措资金，争取新上项目，大幅拉开城市框架，配套完善基础设施，提升城市建设品位。吴忠市区仍然按照建设宁夏次中心城市的目标，高起点、高品位规划，大手笔、大气魄建设。以优化布局、完善功能、美化环境为指导改造提升老城，以只争朝夕的精神加快开发建设新区。重点抓好“民居计划”、新区广场等10项重点工程，办好建设宣传文化中心、疾病控制中心等10件大事，新建中央大道、中水回用等项目，把新区建设成充满现代气息的行政区域中心，成为吴忠市区一道亮丽的风景。加大“城中村”改造力度，消除不利于城市发展的体制和政策障碍，改革户籍管理制度，完善配套设施，鼓励农民进城进镇，聚集城市人口、扩张城市容量。大力发展第三产业。以旅游、商贸流通、物流配送、金融电信、市政服务、文化娱乐、餐饮等为重点，提升第三产业发展水平。深入整顿和规范房地产市场秩序，促进房地产业健康发展。精心打造旅游品牌，在提高沙坡头等旅游景区品位的同时，加快古城湾、罗山、盐池哈巴湖等新景区开发建设，增强旅游对第三产业的拉动力。加强基础设施建设。配合搞好扶贫扬黄灌溉工程、沙坡头水利枢纽工程和中郝、中固高速公路建设。抓住国家实施西部通县油路建设的机遇，加快城市道路和县乡村公路建设，建成吴青公路。实施城乡电网改造及公众电信网、广播电视网建设，支撑经济发展。

(三)用抓工业的理念发展农业，努力增加农民收入。

建设现代农业、发展农村经济、增加农民收入是全面建设小康社会的重大任务。加快推进农业产业化，是实现我市经济跨越式发展的基础，必须按照发展现代农业的要求，强化市场意识和效益观念，用工业的经营理念、经营方式发展农业；保护和扩大我市现代农业在全区领先发展的优势，以构建特色农业产业体系为目标，以培育优质无公害农产品为重点，以提升产业化水平为主导，全面深化农业和农村经济结构调整。抓龙头强农。坚持一县重点发展一个农业产业化龙头企业，带动一个支柱产业的思路，大力发展农副产品加工、运销储藏企业，重点扶持夏进奶业、香山酒业、天启薯业、绿海草业等产业化龙头企业和中宁枸杞、中卫禽蛋等农产品批发市场。充分发挥企业与市场在农业结构调整中的主导作用，吸引大公司参与产业化经营；加强农业社会化服务体系建设，提高农业市场化和组织化程度，促进贸工农联接和产加销衔接，延长产业链，提高附加值。大力发展农村二三产业，推进乡镇企业二次创业，培育扩大一乡一业、一村一品的产业发展格局，引导农民发展非农产业，拓宽农民增收的渠道。抓科技兴农。深入落实科技兴农的各项措施，增强园区的辐射带动作用，加强农民技术培训，加速技术转化应用；引导农民由吨粮田建设向千元田、万元田转变。用工厂化管理和先进实用技术提升特色优势产业，积极发展标准化农业、高品质农业，发展绿色食品、无公害食品，满足消费市场的更高要求。抓畜牧富农。把畜牧业作为结构调整和农民增收的一个大产业，加快品种改良繁育和育肥出栏，优先扶持奶产业、羊产业、设施养殖业扩张总量和规模，巩固发展家禽和生猪产业，进一步提高畜牧业在农业产值和农民收入中的比重。充分发挥区域优势，聚合生产要素，努力做强做优“三白一红一绿”产业，提高综合效益。抓生态促农。抓住自治区实施中部干旱带生态治理的契机，不失时机地建设生态吴忠。全面实施中部干旱带封山禁牧和退耕还林、“三北”防护林、天然林保护工程，加快生态环境治理。加强领导，强化措施，确保五月一日前全面禁牧，确保禁牧不反弹。增加对农业基础设施的投入，积极应对今年灌区灌溉用水大幅减少的严峻形势，继续加强农田水利基本建设、灌区节水改造、农业综合开发和山区“生命工程”建设，改善农业生产条件。抓扶贫攻坚。把盐池、同心、红寺堡等贫困区的发展摆到突出位置，深入实施“千村扶贫开发工程”、“十万贫困户养羊工程”、“十万户沼气工程”和贫困村扶贫规划，加大扶贫投入，推进社会扶贫，增强贫困地区的自我发展能力。积极争取扶贫项目和投资，扩大劳务输出，多渠道增加农民收入，为稳定解决温饱打好基础。全面落实农村税费改革试点的政策措施，切实减轻农民负担。

(四)创新环境，深化开放，扩大投资。

发展环境就是生产力。在市场环境更趋成熟的今天，没有好的环境，就很难在争取项目、争取投资上有大的作为，就难以培育新的竞争优势和发展动力。面对周边地区咄咄逼人的竞争态势，面对各地争先恐后的发展势头，我们必须把优化和创新经济发展环境摆上更加紧迫、更加突出的位置，坚定不移地按照自治区创新经济发展环境的要求和部署，积极适应市场经济和对外开放的新要求，从改进管理和服务经济的方式、优化法治环境、完善政策措施、提高人文素质等方面入手，创新市域经济发展环境。着力从体制、机制上加快向发达地区和国际管理规则接轨，努力减少行政、体制等非经济因素对经济发展的制约，营造有利于开放、有利于发展的氛围。继续实施开放带动和项目带动战略，全力以赴抢项目、争投资。紧紧抓住国家继续实施积极的财政政策和对西部地区实施政策、资金倾斜的机遇，动员各方面力量关注和研究国家在产业、环保、教育、科技、生态等方面的投资政策，筛选储备项目，积极争取国家、自治区支持。继续深化对外开放，深化鲁宁、闽宁合作，落实好已签约项目，争取新的招商引资项目。争取金融支持，吸引客商参与企业技术改造、城市建设和交通通信开发。鼓励有实力的企业走出去谋求发展，引进技术和资金。积极发展对外贸易，扩大出口规模。认真落实对外开放的政策措施，依法保护投资者的合法权益，努力创造一个诚实守信、有序竞争的投资环境，提高对外开放水平。

(五)加强财政税收和审计工作，支持经济与社会事业发展。

大力培植和开拓财源，以经济发展促进财政增收。注重和加强市本级财源建设，巩固税收基础，提高市本级财政调控能力。大力整顿税收秩序，强化税收征管，堵塞税收漏洞，依法清缴欠税，做到依法治税、依率计征，确保税收收入的稳定增长。积极推进财政体系改革，推行国库集中收付制度，扩大政府采购范围，加强“收支两条线”管理。按照“一要吃饭、二要建设”的原则，坚持从严从紧管理财政支出，进一步优化支出结构，在保证刚性支出的同时，努力增加农业、科技、教育、文化、卫生、体育、社会保障等事业支出。强化审计监督和财政管理，整顿和规范财经秩序，严肃财经纪律，提高财政资金使用效益，不断强化财政在经济运行和社会发展中的保障作用。

三、切实加强精神文明和民主法制建设，推动社会全面进步

始终坚持“两手抓、两手都要硬”的方针，贯彻依法治市、以德治市方略，不断加强精神文明和民主法治建设，促进社会事业全面发展。

(一)大力推进精神文明建设。按照新的时代要求，坚持用马列主义、毛泽东思想、邓小平理论、“三个代表”重要思想和党的十六大精神教育广大干部群众，凝聚发展动力。紧贴社会实际，广泛进行爱国主义、集体主义、社会主义思想教育；加强社会公德、职业道德、家庭美德教育，营造良好的社会风尚。以群众喜闻乐见的形式，广泛开展文明创建活动，开展“青年志愿者”、“希望工程”、“扶残助残”、“五好文明家庭”等活动。深化科普教育，弘扬科学精神，反对各种错误思潮和封建迷信。加强全民国防教育和双拥工作，密切军政、军民关系。

(二)坚持走可持续发展道路。坚持不懈地做好人口与计划生育工作，强化人口与计划生育目标管理责任制，实施“少生快富工程”，促进优生优育。建立健全土地执法监察制度、收购储备制度和有偿使用制度，推行土地使用权、矿业权公开招标、拍卖及挂牌出让。加强城市土地一级市场管理，规范征用程序和交易秩序，盘活存量土地。严格执行土地利用总体规划，合理利用和保护耕地，保持耕地占补总量平衡。加大环境保护力度，强化执法监督和综合治理，严格排污总量控制，实现工业污染物达标排放。坚持建设项目预审和环保评价制度，严格控制新污染源产生。

(三)全面推进科教兴市。加快先进技术和科技成果的转化和应用，支撑农业，提升工业，提高产品科技含量。积极推行科技特派员制度，引导科研机构和科技人才进企业、进园区，示范推广新技术。深化教育体制改革，全面推进素质教育，调整基础教育结构，，扩大高中，优化中小学布局。川区巩固提高“两基”成果，山区加快“普九”进程。继续推进民族教育、职业教育健康发展，加快中小学教育信息化建设，发展现代远程教育。实施“人才战略”，加强人才培养和人才资源开发。落实知识、技术、管理等生产要素参与分配的政策，充分调动各类人才的积极性和创造性。

(四)积极发展社会事业。发展时代精神与地方特点相融合的先进文化。大力开展形式多样的城乡文化活动，丰富群众的文化生活。办好报刊和广播电视。切实加强文物保护和管理。广泛开展全民健身活动，发展竞技体育，积极筹备参加第七届全国少数民族传统体育运动会。深化城乡卫生体制改革，建立和完善医疗服务要素准入制度和行业自律机制，提高医疗服务水平。切实保护妇女儿童和残疾人的合法权益，促进老龄和残疾人事业健康发展。

(五)健全和完善社会保障体系。从维护改革发展稳定的大局出发，完善社会保障制度建设。继续深入落实“两个确保”政策，采取有效措施，加大社保基金征缴、清欠力度，加强社保资金的监督管理，确保国有企业下岗职工基本生活费和离退休人员养老金按时足额发放。进一步扩大社会保险覆盖面，全面推进县(市、区)医疗保险制度改革。认真落实城市居民最低生活保障制度和农村贫困人口最低生活救助制度，保障城乡低收入群体的基本生活。切实做好就业和再就业工作，进一步落实促进就业和再就业的优惠政策，帮助、鼓励下岗职工、失业人员多渠道就业。积极发展社会福利、社会经济、优抚安置等社会保障事业，不断完善社会保障网络。

(六)加强民主法制建设。自觉接受人大及其常委会的法律监督和政协的民主监督，密切与各民主党派、人民团体和社会各界的联系，认真听取各方面的意见和建议，积极改进政府工作。推进依法行政，加强政府法制建设，健全执法责任制，全面推行政务公开、村务公开、厂务公开，加强基层民主政治建设。继续深化依法治市，深入开展“四五”普法教育，提高公民的法律素质，促进社会法治化管理。认真贯彻执行《民族区域自治法》，依法管理宗教事务，巩固和发展平等、团结、互助的社会主义新型民族关系。

(七)全力维护社会稳定。牢固树立稳定压倒一切的思想，重视和加强信访工作，正确处理和及时化解社会矛盾和纠纷，把各种不安定因素解决在萌芽状态。层层落实社会治安综合治理责任制，建立健全社会治安防范网络。坚持打防并举、标本兼治，深入开展严打整治专项斗争，依法打击各种刑事犯罪和恶势力，全力扫除黄、赌、毒等社会丑恶现象，保持社会稳定。

四、模范实践“三个代表”重要思想，提高政府管理经济服务社会的效能

坚持以党的十六大精神为指导，以建立行为规范、运转协调、公正透明、廉洁高效的行政管理体制为目标，模范实践“三个代表”重要思想，建设责任政府、法制政府、服务政府，提高政府管理经济、服务社会的效能。

(一)不断优化政府工作职能。继续深入推进行政管理体制改革，促进政府管理创新，改进政府管理经济的方式，充分发挥市场配置资源的基础作用和经济杠杆的调节作用，综合运用市场的、法律的和经济的手段管理经济。积极推进依法行政，加快行政审批制度改革，减少审批事项，简化审批程序，规范审批行为。继续推行政务公开，规范和完善市政府政务大厅管理制度和办事制度，增强政府工作的透明度，加强监督管理，提高办事效率。推进事业单位人事制度改革。大力倡导深入实际、务实求进的工作作风，明确责任，统筹协调，狠抓落实，全力推进各项工作。

(二)努力提高管理服务经济的水平。引导政府各部门积极进取，高效工作，提高管理和服务经济的能力，提高科学决策和依法行政水平。创新工作思路和方式，克服旧的思维模式，创造性地开展工作。深入调研，科学决策，研究处理好经济和社会发展中的新情况和新问题，更多地政策引导、市场监管、法治保障等方面加强服务，确保经济和社会事业顺利推进。健全和实施科学的目标责任制和工作监督制度，在政府机关实行首问责任制、限时办理考核和行政首长负责制，加大对重大决策、重点工作、重要项目、客商投资服务的督查和责任追究，对在年度工作测评考核中处于末位的进行诫免，实行末位淘汰。深入贯彻落实中纪委第二次全体会议精神，全面落实党风廉政建设责任制，加强对各级干部的廉政教育，进一步完善监督和防范机制，规范领导干部从政行为，深化纠正部门和行业不正之风，从源头上预防和治理腐败。

(三)努力建设高素质的公务员队伍。以邓小平理论和“三个代表”重要思想、党的十六大精神为主要内容，加强对政府公务员的理论教育和市场经济、现代科技、WTO知识、法律等知识的培训，提高理论水平、知识层次和工作能力，牢固树立爱岗敬业、廉洁奉公、遵纪守法的良好风范。紧密联系公务员思想实际和工作实际，联系我市全面建设小康社会的目标、任务，增强公务员加快发展的危机意识和责任意识，动员各级公务员以积极进取的精神风貌、扎实有效的作风，集中精力推进工作落实，加快全市经济和社会发展。

各位代表，加快推进全面建设小康社会的进程，实现吴忠经济的更大发展和社会的繁荣进步，任务光荣而艰巨。让我们高举邓小平理论伟大旗帜，努力实践“三个代表”重要思想，全面贯彻党的十六大精神，与时俱进，开拓创新，为确保完成今年的各项任务，为实现吴忠改革开放和现代化建设的新跨越而努力奋斗！
